# GLP1R rs3765467 Polymorphism Is Associated with the Risk of Early Onset Type 2 Diabetes

**DOI:** 10.1155/2023/8729242

**Published:** 2023-12-14

**Authors:** Yunyun Fang, Jingjing Zhang, Linlin Ji, Chaoyu Zhu, Yuanyuan Xiao, Qingge Gao, Wenjing Song, Li Wei

**Affiliations:** ^1^Department of Endocrinology and Metabolism, Shanghai Jiao Tong University Affiliated Sixth People's Hospital, Shanghai Diabetes Institute, Shanghai Key Laboratory of Diabetes Mellitus, Shanghai Clinical Center for Diabetes, Shanghai Key Clinical Center for Metabolic Disease, 600 Yishan Road, Shanghai 200233, China; ^2^National Demonstration Center for Experimental Fisheries Science Education, Shanghai Ocean University, 999 Hucheng Huan Road, Lingang New City, Shanghai 201306, China

## Abstract

**Objective:**

To investigate the relationship between *glucagon-like peptide-1 receptor* gene polymorphisms and susceptibility to early onset type 2 diabetes.

**Methods:**

Samples from 316 type 2 diabetes patients with early onset type 2 diabetes (*n* = 137) and late-onset type 2 diabetes (*n* = 179) and 145 nondiabetic individuals were analyzed. Multiplex PCR combined with resequencing Hi-Reseq technology was used to detect single nucleotide polymorphisms of the glucagon-like peptide-1 receptor gene, and the allele frequency, genotype distribution, and clinical parameters were analyzed between each diabetes subgroup and the control group.

**Results:**

Sixteen single nucleotide polymorphisms were identified in the exonic region of the glucagon-like peptide-1 receptor gene according to the minor allele frequency (MAF > 0.05) in the participants. Among these, the glucagon-like peptide-1 receptor rs3765467 (G⟶A) mutation was statistically associated with early onset type 2 diabetes. Compared with that of the GG carriers, carriers of genotype AA at rs3765467 had a decreased risk of early onset type 2 diabetes after adjusting for sex and body mass index. In the dominant model, the frequencies of the rs3765467 AA + GA genotype were significantly decreased in the early onset type 2 diabetes group, and carriers of genotype AA + GA at rs3765467 had a decreased risk of early onset type 2 diabetes after adjusting for sex and body mass index. Moreover, fasting C peptide levels were significantly higher in GA + AA genotype carriers than those in GG genotype carriers.

**Conclusion:**

The glucagon-like peptide 1 receptor rs3765467 polymorphism was significantly associated with age at type 2 diabetes diagnosis and thus may be used as a marker to screen and detect individuals at risk of developing early onset type 2 diabetes.

## 1. Introduction

Type 2 diabetes (T2DM) is a chronic metabolic disease characterized by hyperglycemia, and the high rates of diabetes-related morbidity and mortality generate enormous economic and social burdens. The prevalence of T2DM has been increasing exponentially and is expected to increase to 439 million by 2030 [1]. Recent evidence suggests that the prevalence of T2DM in young adults is markedly increasing worldwide [2, 3]. In China, clinical statistical data show that T2DM diabetes is prevalent among people older than 20 years, with the percentages being 15.5% [4]. Several studies have found that early onset type 2 diabetes (EOD, onset age <40 or 45 years) is likely to be associated with an increased prevalence of complications later [5, 6]. The prevalence of EOD has increased more rapidly than that of late-onset type 2 diabetes (LOD), reaching 6.4% [7]. Studies have shown that patients with EOD have an increased magnitude of *β*-cell dysfunction, risk of obesity, and diabetes‐related complications compared with those in LOD and nondiabetic subjects [8]. Thus, it is necessary to explore the underlying risk factors and clinical characteristics of EOD.

Genetics and environmental factors play crucial role in the etiology of diabetes [9, 10]. Study shows that SNPs in the 3′-untranslated region of *SLC30A8* increased the risk of T2DM [11]. Epidemiological evidence indicates that EOD exhibits a tendency of familial aggregation [12, 13]. Recently, a large number of single nucleotide polymorphisms (SNPs) associated with the risk of T2DM have been discovered, but only a few studies have examined the influence of such genetic heterogeneity on the age of onset of T2DM [14–16]. The loss of insulin release, insulin resistance, and increased glucagon secretion also accelerate the development of T2DM. Glucagon-like peptide 1 (GLP1) is an incretin hormone that regulates blood glucose homeostasis by stimulating insulin secretion, increasing insulin sensitivity, delaying gastric emptying, reducing liver glycogen output, and promoting lipolysis of adipose tissue [17, 18]. Glucagon-like peptide 1 receptor (*GLP1R*) is a member of the B1 family of G protein-coupled receptors and is localized to the long arm of chromosome 6 (chr 6p21) in humans. GLP1 specifically targets GLP1R. Previous studies have shown that *GLP1R* polymorphisms are associated with susceptibility to diabetes and clinical responsiveness to different hypoglycemic agents [19]. Moreover, *GLP1R* polymorphisms are associated with lipid metabolism and the risk of coronary artery disease in T2DM patients [20]. However, the relationship between *GLP1R* gene polymorphisms and the onset age of T2DM has not yet been established.

In this study, multiplex PCR combined with high-throughput sequencing was used to detect the distribution of genetic polymorphisms in the exonic region of the *GLP1R* gene. Furthermore, we investigated the association between variations in *GLP1R* and susceptibility to EOD.

## 2. Materials and Methods

### 2.1. Patients

A total of 316 T2DM and 145 nondiabetic control subjects were enrolled in this study from 2018 to 2019 at the Shanghai Sixth People's Hospital in China. Patients with diabetes were diagnosed according to the WHO-2021 diagnostic criteria. Individuals with type 1 diabetes, gestational diabetes, malignancies, systemic inflammation, severe liver disease, chronic kidney disease of stages 3–5, and cardio-cerebrovascular disease were excluded from the study. According to the American Diabetes Association criteria for T2DM screening in adults, patients with T2DM were divided into two subgroups: (1) EOD (*n* = 137; age at diagnosis ≤45 years) and (2) LOD (*n* = 179; age at diagnosis >45 years).

### 2.2. Anthropometric and Biochemical Measurements

A questionnaire for collecting data on age, history of diabetes, medications, and family history was completed in a face‐to‐face interview. Resting blood pressure was measured. Body mass index (BMI) was calculated as the weight (kg) divided by the square of height (kg/m^2^).

A radioimmunoassay was used to measure the levels of serum fasting insulin (FINS), 2 h postmeal insulin (2 h INS), fasting C peptide (FCP), and 2 h postprandial CP (2 h CP). The homeostasis model assessment for insulin resistance (HOMA-IR) and for islet *β*-cell function (HOMA-*β*) was used to calculate these values [21]. Hemoglobin A1c (HbA1c), glycated albumin (GA), fasting plasma glucose (FPG), 2 h postprandial blood glucose (2 h-PG), triglyceride (TG), total cholesterol (TC), low-density lipoprotein (LDL), and high-density lipoprotein were measured using an automated bioanalyzer (Rayto Technologies, China).

### 2.3. DNA Extraction and Direct Resequencing of the GLP1R Gene

Cubital venous blood was collected from all subjects and stored in a 5 mL EDTA anticoagulant tube at −20°C. Whole blood genomic DNA was extracted with a blood genomic DNA extraction kit (Lifefeng Biotech Co. Ltd., China), and the quality of DNA samples was measured using an Enzyme Marker (SpectraMax I3x) to ensure that the concentration was ≥50 ng/L. The absorbance value at A260/A280 was between 1.8 and 2.0. The extracted DNA samples were stored at −80°C.

Multiplex PCR-specific primers for the GLP1R gene were designed according to the DNA reference sequence of humans in NCBI. The extracted DNA samples were used as templates for PCR amplification. The different samples were identified by corresponding barcode primers, and then the amplicons were sequenced by second-generation high-throughput sequencing (Illumina HiSeq X Ten). Sequencing results were analyzed using bioinformatics methods, and the variation information of each site was obtained.

To control against false-positive SNPs, FastQC was used to filter out low-quality sequences, and the retained data sequencing quality *Q* was> 20, which indicates an accuracy rate of 99%. Before sequence alignment, Cutadapt software was used to remove the sequences added to both sides of the specific primers. Burrows-Wheeler Aligner was used to map the second-generation sequencing short segments to the reference genome (GRCh37/HG19). Then, GATK software was used for the statistical analysis of SNP sites in sequencing fragments. Finally, the mutation information was filtered based on the following criteria: sequencing depth at the mutation site >30 and occurrence of mutation at least once in all samples.

### 2.4. Statistical Analysis

Normally distributed quantitative data are expressed as $\bar{x}$ ± *s*, and data with skewed distribution are expressed as medians (interquartile range). The Hardy–Weinberg equilibrium (HWE) was investigated by Pearson's test. Nonconditional logistic regression analysis was performed with genotypes, alleles, dominant and recessive models, and odds ratios (OR), and 95% confidence intervals (95% CI) were calculated. *χ*^2^test, independent sample *t*-test, and nonparametric tests were used to analyze the changes in clinical parameters among the control, EOD, and LOD groups or between the different genotype groups. One-way analysis of variance was performed for comparisons between multiple groups. To avoid statistical errors resulting from nonparametric tests, we calculated the HOMA-IR and HOMA-*β* values for the related parameters, thereby converting them into normal distributions, and then performed parametric tests. All statistical analyses were performed using SPSS software version 22.0, and *P* values <0.05 were considered significant.

## 3. Results

### 3.1. Clinical Characteristics

The EOD group had a higher percentage of male subjects than the control group; otherwise, no significant differences were found in sex between the control and LOD groups. Moreover, significant differences in BMI, TG, HbA1c, FPG, HOMA-IR, and HOMA-*β* were identified for each diabetes subgroup in comparison with those in the control group. However, no differences were found in 2 h insulin, TC, and LDL between each diabetes subgroup and control group (Table 1).

### 3.2. Allele and Genotype Analysis

**Figure 1:**
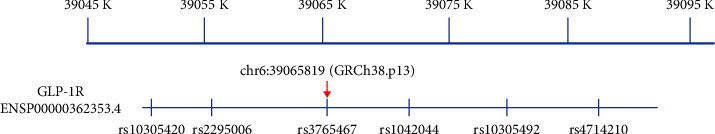
The positions of rs3765467 in *GLP1R* gene.

Sixteen SNPs in the exonic region of the *GLP1R* gene were identified according to the minor allele frequency (MAF > 0.05) in the participants (Table 2). Among these 15 SNPs, only polymorphisms at position rs3765467 in the *GLP1R* gene were statistically associated with EOD (*P* < 0.001). The genotype distributions of *GLP1R* rs3765467 in each group achieved HWE (*P* > 0.893), indicating that no factors could significantly affect the genetic balance in our study population, which is suitable for population genetics research. The rs3765467 G/A polymorphism induces amino acid sequence changes in *GLP1R*, as arginine at position 131 was replaced with glutamine (HGVSp ENSP00000362353.4: p.A rg131Gln) (Figure 1).

**Figure 2:**
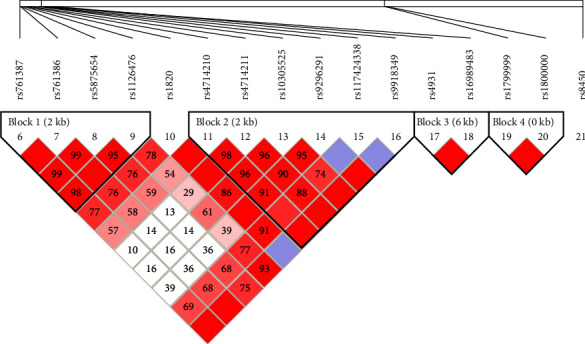
Chain transfer imbalance map of single nucleotide polymorphism of glucagon-like peptide-1 receptor gene.

The linkage disequilibrium analysis was performed by Haploview. Result showed no linkage disequilibrium between rs3765467 and other sites (Figure 2). In allelic frequency analysis, the frequency of the A allele at position rs3765467 was significantly decreased in patients with EOD compared with that in nondiabetic individuals, with an OR of 0.410 (95% CI: 0.262–0.642) (Table 3). In genotype association tests, carriers of genotype AA at rs3765467 had a decreased risk of EOD (OR = 0.244, 95% CI = 0.079–0.750, *P*=0.014) after adjusting for sex and BMI. In the dominant model, the distribution of the rs3765467 AA + GA genotype was significantly decreased in the EOD group, and carriers of genotype AA + GA at rs3765467 had a decreased risk of EOD (OR = 0.365, 95% CI = 0.204–0.655, *P* < 0.001) after adjusting for sex and BMI. However, no significant association was observed for the *GLP1R* rs3765467 variant in the recessive model. Moreover, the *GLP1R* rs3765467 variant displayed similar allele frequencies and genotype distributions between the LOD group and control group, and no significant associations were observed between genotype and LOD risk.

Heritability is used to describe the proportion of genetic variation in phenotypic variation. Phenotype (P) is jointly controlled by genotype (G) and environmental factor (E), that is, heritability is the proportion of gene G. Specifically, genetic variation and phenotypic variation are described by variance. Numerator was used as the variance of a set of sample genotypes and the denominator as the variance of phenotypes. There were 95 patients with early diabetes caused by genotype and 221 patients with diabetes affected by environmental factors in this study. The calculation gives a heritability of 0.7 (Table 4).

### 3.3. Clinical Characteristics according to *GLP1R* Genotypes

As shown in Table 5, we further analyzed the clinical characteristics according to rs3765467 genotype in a dominant model after subgrouping of the EOD group. Results revealed no significant differences between GA + AA and GG groups in sex, age, BMI, fasting plasma glucose, HbA1c, FINS, HOMA-IR, and HOMA-*β*. Interestingly, FCP levels were significantly increased in patients with GA + AA genotype (*P* < 0.042), compared with those in patients with the GG genotype. However, all clinical characteristics were similar for patients with the GG and GA + AA genotypes in the LOD group (Table 6).

## 4. Discussion

Recently, the relationship between *GLP1R* polymorphisms and T2DM was reported to vary with ethnicity [22]. However, current studies mainly focus on whether *GLP1R* gene variants increase the risk of T2DM without age stratification [23]. The prevalence of EOD has increased rapidly worldwide, especially in Asian countries [24]. In this study, we investigated the relationship between genetic variation of the *GLP1R* gene and susceptibility to EOD.

We defined adult T2DM patients diagnosed at age ≤45 years as having EOD. Consistent with the results of the 2010 General Survey of Diabetes in China [25], this study found that blood glucose, blood lipid, BMI, insulin resistance, and other indexes in EOD patients were significantly higher than those in nondiabetic subjects. In the sex analysis, the proportion of male patients in the EOD group was significantly higher than that of female patients (*P* < 0.01), which may be related to the complex interaction between genes and the environment. Recently, gene variants, such as TRIB3 (rs2295490), ADIPOQ (rs10937273), LEPR (rs1892534), and TCF7L2 (rs7903146), were identified as risk factors for EOD [16, 26–28]. *GLP1R* is a G-protein-coupled receptor with a typical seven-transmembrane *α*-helical core domain (TMD) and an extracellular domain (ECD), playing a critical role in hormone signal transduction and receptor activation. Once the ligand contacts the receptor, the ECD of *GLP1R* firstly binds to the C-terminal of the ligand, followed by binding of the N-terminal of the ligand to the TMD of the receptor, thus activating the receptor. Amino acid residues in the extracellular domain of *GLP1R* play a critical role in its self-folding and structural stability [29]. Previous studies have reported that *GLP1R* gene polymorphisms are closely related to insulin secretion and response to *GLP1R* analogs in diabetes patients. In hyperglycemic clamp experiments in healthy individuals, Sathananthan showed that rs3765467 was associated with altered *β*-cell response to GLP1 infusion [30]. Moreover, the rs6923761 polymorphism of the *GLP1R* gene is closely related to fasting serum levels of GLP1 and adipocytokine in newly diagnosed T2DM patients [31]. In addition, carriers of genotype GG at rs4714210 in *GLP1R* showed a decreased risk of coronary heart disease [32]. However, the correlation between *GLP1R* gene polymorphism and the onset age of T2DM, especially EOD, has not been reported. This study is the first to investigate *GLP1R* gene polymorphisms and their effect on clinical characteristics in a Chinese EOD population. We found that among the 16 SNP sites with MAF > 0.05, only the polymorphism at position rs3765467 in *GLP1R* was statistically associated with EOD. Previous studies have indicated that the most frequent substitution in *GLP1R* is Gly-168 to Ser (G⟶A, rs6923761) [20]. However, the MAF of rs6923761 was <0.01 in this study, which might be attributed to the limited samples used and ethnic differences; thus, statistical analysis of the data could not be carried out.

The rs3765467 polymorphism involves an Arg-131 to Gln (G⟶A) substitution, which affects the binding efficiency of the ligand and *GLP1R*, thereby interfering with downstream signal transduction. Dipeptidyl peptidase 4 (DPP-4) inhibitors are common hypoglycemic drugs that can prevent the degradation of GLP1. Polymorphism at position rs3765467 in *GLP1R* may influence response to DPP-4 inhibitors. Patients with the rs3765467 GA + AA genotype had a relatively better response to DPP-4 inhibitors and a greater reduction in HbA1c [33]. Another study found that the *GLP1R* rs3765467 G > A variant could significantly reduce insulin secretion and cyclic AMP (cAMP) concentration in response to high glucose exposure, which might promote *β* cell apoptosis [34]. In this study, carriers of allele A at rs3765467 were independently associated with a risk of EOD after adjusting for sex and BMI. Furthermore, compared with that of the GG genotype, the GA + AA genotype decreased the risk of EOD, further confirming that the rs3765467 polymorphism is closely related to susceptibility to EOD. However, neither the distribution of the genotype nor the allelic frequency of rs3765467 was statistically different between the LOD and nondiabetic population. We speculated that the presence of the A allele at position rs3765467 may be specifically associated with a reduced susceptibility to EOD.

Previous studies have shown that patients with EOD suffer from a more severe impairment of islet *β*-cell function and require insulin treatment at an earlier time [35]. In this study, the effect of genotypes on clinical characteristics among EOD patients was also investigated. No significant differences were found in HOMA-IR and HOMA-*β* between rs3765467 GG and GA + AA genotype carriers. Studies show that the association between these genotypes and insulin levels could not be assessed accurately by HOMA-IR and HOMA-*β*, as most patients had been treated with hypoglycemic agents and/or insulin. Herein, we also assessed the levels of CP, a marker of insulin secretion, between these genotypes. Compared with those of the rs3765467 GG genotype carriers, patients with the GA + AA genotype showed increased FCP levels in the EOD group. Inconsistently, the fasting plasma glucose was similar for patients with the GG and GA + AA genotypes. We speculated that the GLP1R rs3765467 (G⟶A) mutation may influence the impaired islet function of *β*-cells in EOD patients; however, the relative small case number might limit the power to detect a difference.

Although this study showed a correlation between *GLP1R* rs3765467 and susceptibility to EOD, it has a few limitations. First, this is a single-center study, and there may be sample selection bias. Second, the rs3765467 polymorphism had no significant effect on other clinical parameters in EOD patients, which may be attributed to the small sample size and our inability to detect weak associations. Third, no association was detected for all SNPs in *GLP1R* between the LOD and control groups. As SNPs with a MAF < 0.01 in the *GLP1R* gene could not be detected, the slight effects of these *GLP1R* variants were also possibly undetected. Therefore, further replications in other cohorts are needed to confirm the association of genetic variants of the *GLP1R* gene with susceptibility to EOD.

## 5. Conclusion

In conclusion, we showed that the *GLP1R* rs3765467 polymorphism in the *GLP1R* gene was significantly associated with the age at diagnosis of T2DM in a Chinese population. The *GLP1R* rs3765467 polymorphism may thus be used as a marker to screen and detect individuals at risk of developing EOD.

## Figures and Tables

**Table 1:** Clinical characteristics of the study population.

	Control	Onset age of T2DM (years)			
		EOD ≤ 45	LOD > 45	*P* ^a^	*P* ^b^
Subjects (*n*)	145	137	179	—	—
Sex (M/F)	58/87	85/52	73/106	<0.001	0.887
Age (years)	53.1 ± 6.6	46.9 ± 10.3	60.3 ± 6.8	<0.001	<0.001
BMI (kg/m^2^)	23.7 ± 3.3	26.0 ± 3.6	25.7 ± 3.8	<0.001	<0.001
Systolic blood pressure (mmHg)	122.6 ± 14.0	132.8 ± 18.1	137.8 ± 20.2	<0.001	<0.001
Diastolic blood pressure (mmHg)	78.1 ± 8.9	77.8 ± 11.1	79.9 ± 13.7	0.972	0.364
TG (mmol/L)	1.1 (0.7–1.8)	1.7 (1.1–3.0)	1.6 (1.1–2.6)	<0.001	<0.001
TC (mmol/L)	4.9 ± 0.9	5.1 ± 1.4	5.1 ± 1.6	0.291	0.263
LDL (mmol/L)	3.1 ± 0.7	3.3 ± 0.8	3.3 ± 0.8	0.107	0.118
HDL (mmol/L)	1.3 ± 0.3	1.0 ± 0.3	1.0 ± 0.3	<0.001	<0.001
HbA1c (%)	5.6 ± 0.3	9.4 ± 2.2	9.6 ± 2.8	<0.001	<0.001
GA (%)	13.2 ± 1.1	23.6 ± 7.6	26.1 ± 16.0	<0.001	<0.001
Fasting plasma glucose (mmol/l)	5.3 ± 0.4	8.8 ± 2.5	8.7 ± 2.3	<0.001	<0.001
2 h glucose (mmol/L)	6.4 ± 1.1	12.2 ± 4.0	13.3 ± 5.3	<0.001	<0.001
Fasting serum insulin (mU/L)	6.5 (4.2–8.7)	10.2 (7.0–15.2)	10.1 (6.0–16.2)	<0.001	<0.001
2 h insulin (mU/L)	30.7 (18.8–51.7)	38.5 (17.7–66.6)	38.3 (21.1–64.1)	0.193	0.082
HOMA-IR	1.5 (1.0–2.1)	3.8 (2.5–5.8)	3.7 (2.2–6.4)	<0.001	<0.001
HOMA-*β*	74.0 (48.6–101.1)	46.4 (26.4–78.2)	41.9 (23.1–75.5)	<0.001	<0.001

*P* ^a^-value for comparison of the EOD and control groups. *P*^b^-value for comparison of the LOD and control groups.

**Table 2:** Allele frequency of *GLP1R* SNPs.

SNP ID	REF	ALT	Control (R/A)	EOD (onset age ≤45 yr) (R/A)	LOD (onset age> 45 yr) (R/A)	*P* ^b^ value
rs3765468	G	A	245/45^a^	218/56^a^	304/54^a^	0.158
rs3765467	G	A	201/89^a^	226/48^b^	268/90^a,b^	<0.001
rs2235868	A	C	156/134^a^	168/101^a^	202/152^a^	0.192
rs1042044	A	C	134/156^a^	142/132^a^	176/182^a^	0.409
rs761387	A	G	239/41^a^	193/47^a^	260/44^a^	0.206
rs761386	C	T	244/42^a^	221/51^a^	305/51^a^	0.269
rs5875654	CAG	C	245/43^a^	218/54^a^	305/51^a^	0.141
rs1126476	A	C	152/138^a^	166/108^a^	200/158^a^	0.145
rs1820	T	A	267/23^a^	251/23^a^	335/23^a^	0.611
rs4714210	A	G	207/81^a^	194/78^a^	253/103^a^	0.978
rs4714211	A	G	178/110^a^	164/110^a^	221/137^a^	0.872
rs10305525	C	A	190/96^a^	179/93^a^	237/119^a^	0.984
rs9296291	T	C	216/70^a^	206/64^a^	278/74^a^	0.547
rs117424338	C	T	268/22^a^	254/20^a^	335/23^a^	0.832
rs9918349	G	A	248/42^a^	238/36^a^	314/44^a^	0.708

REF: the base of the reference genome; ALT: the bases of a mutant genome; R/A: the ratio of the number of REF to that of ALT. *P*^b^-value for comparison of the EOD and control groups. ^a^No significant difference between the EOD group and control group and between the LOD group and control group. ^b^Significant difference between the EOD group and control group. ^a,b^Significant difference between the LOD group and control group.

**Table 3:** Association between *GLP1R* rs3765467 polymorphism and susceptibility to diabetes.

	Control	Onset age of T2DM (years)		Control vs. EOD		Control vs. LOD	
		≤45	>45	OR (95% CI)^a^	*P* ^a^	OR (95% CI)^b^	*P* ^b^
*Allele*							
G	201 (69.3%)	226 (82.5%)	268 (74.9%)	1		1	
A	89 (30.7%)	48 (17.5%)	90 (25.1%)	0.410 (0.262∼0.642)	<0.001	0.775 (0.534∼1.123)	0.178

*Genotype*							
GG	70 (48.3%)	95 (69.3%)	105 (58.7%)	1		1	
GA	61 (42%)	36 (26.3%)	58 (32.4%)	0.365 (0.204∼0.655)	0.001	0.679 (0.409∼1.129)	0.136
AA	14 (9.7%)	6 (4.4%)	16 (8.9%)	0.244 (0.079∼0.750)	0.014	0.742 (0.326∼1.690)	0.477

*Dominant model*							
GG	70 (48.3%)	95 (69.3%)	105 (58.7%)	1		1	
GA + AA	75 (51.7%)	42 (30.7%)	74 (41.3%)	0.365 (0.204∼0.655)	<0.001	0.679 (0.409∼1.129)	0.136

*Recessive model*							
GG + GA	131 (90.3%)	131 (95.6%)	163 (91.1%)	1		1	
AA	14 (9.7%)	6 (4.4%)	16 (8.9%)	0.667 (0.209∼2.127)	0.494	1.092 (0.467∼2.554)	0.839

OR, odds ratio; CI, confidence interval. Nonconditional logistic regression was used to calculate ORs, CIs, and corresponding *P* values. OR^a^, 95% CI^a^, and value for comparison of the EOD and control groups (adjust for sex and BMI) . OR^b^, 95% CI^b^, and value for comparison of the LOD and control groups (adjust for sex and BMI).

**Table 4:** Genetic analysis.

Genotype	Types of diabetes	Patients	Assignment
GG	EOD	95	1
	LOD	105	0

GA + AA	EOD	42	0
	LOD	74	0

**Table 5:** Comparison of clinical characteristics according to rs3765467 genotype after subgrouping the EOD group.

Parameters	GG	GA + AA	*P*
Subjects (*n*)	95	42	—
Sex (M/F)	63/32	22/20	0.121
Age (years)	47.2 ± 10.0	46.3 ± 11.1	0.611
Duration (years)	9.6 ± 7.7	8.9 ± 8.4	0.471
BMI (kg/m^2^)	25.7 ± 3.6	26.6 ± 3.6	0.189
Systolic blood pressure (mmHg)	132.8 ± 17.6	133.3 ± 19.6	0.870
Diastolic blood pressure (mmHg)	77.6 ± 11.1	78.5 ± 11.3	0.677
TG (mmol/L)	1.8 (1.3–3.0)	2.1 (1.2–3.8)	0.372
TC (mmol/L)	5.1 ± 1.1	5.1 ± 2.0	0.816
LDL (mmol/L)	3.2 ± 0.8	3.3 ± 0.99	0.551
HDL (mmol/L)	1.0 ± 0.3	1.0 ± 0.24	0.648
HbA1c (%)	9.3 ± 2.3	9.5 ± 2.1	0.677
GA (%)	24.2 ± 7.9	22.4 ± 6.8	0.232
Fasting plasma glucose (mmol/l)	8.7 ± 2.4	9.0 ± 2.6	0.614
2 h glucose (mmol/L)	11.9 ± 4.0	12.8 ± 3.9	0.256
Fasting serum insulin (mU/L)	9.4 (6.2–15.3)	11.8 (7.8–15.2)	0.381
2 h insulin (mU/L)	37.4 (14.6–65.6)	33.3 (21.3–52.9)	0.882
FCP (ng/mL)	1.7 (1.1–2.4)	2.3 (1.5–2.8)	0.042
30 min CP (ng/mL)	2.2 (1.4–3.3)	2.7 (2.0–3.3)	0.066
2 h CP (ng/mL)	3.0 (1.7–4.5)	3.6 (2.4–5.7)	0.170
HOMA-IR	3.5 (2.4–5.6)	4.5 (2.8–6.6)	0.454
HOMA-*β*	47.9 (23.8–71.4)	35.6 (28.5–83.6)	0.879
HOMA-IR^*∗*^	1.3 ± 0.8	1.4 ± 0.7	0.497
HOMA-*β*^*∗*^	3.7 ± 0.8	3.9 ± 0.8	0.280

^*∗*^Natural logarithm.

**Table 6:** Comparison of clinical characteristics according to rs3765467 genotype after subgrouping the LOD group.

Parameters	GG	GA + AA	*P*
Subjects (*n*)	105	74	—
Sex (M/F)	42/63	31/43	0.800
Age (years)	60.1 ± 7.0	60.5 ± 6.7	0.707
Duration (years)	6.7 ± 5.7	6.8 ± 6.0	0.962
BMI (kg/m^2^)	25.8 ± 3.7	25.6 ± 4.0	0.681
Systolic blood pressure (mmHg)	138.8 ± 18.3	136.6 ± 22.5	0.498
Diastolic blood pressure (mmHg)	81.2 ± 14.0	78.1 ± 13.2	0.154
TG (mmol/L)	1.9 (1.3–2.8)	1.8 (1.2–3.2)	0.863
TC (mmol/L)	5.2 ± 1.7	4.9 ± 1.6	0.251
LDL (mmol/L)	3.3 ± 0.8	3.2 ± 0.8	0.548
HDL (mmol/L)	1.1 ± 0.3	1.1 ± 0.3	0.936
HbA1c (%)	9.6 ± 3.0	9.7 ± 2.5	0.913
GA (%)	26.6 ± 19.4	25.5 ± 9.6	0.694
Fasting plasma glucose (mmol/l)	8.5 ± 2.4	8.9 ± 2.1	0.267
2 h glucose (mmol/L)	13.2 ± 4.8	13.4 ± 6.0	0.766
Fasting serum insulin (mU/L)	9.8 (6.2–16.4)	10.5 (5.6–16.2)	0.950
2 h insulin (mU/L)	38.6 (21.1–57.7)	37.9 (20.1–70.6)	0.793
FCP (ng/mL)	1.9 (1.4–2.8)	2.0 (1.4–3.0)	0.830
2 h CP (ng/mL)	4.3 (2.5–7.0)	4.3 (2.1–6.7)	0.711
HOMA-IR	3.5 (2.1–5.6)	3.9 (2.3–6.6)	0.594
HOMA-*β*	41.1 (25.9–85.6)	39.8 (19.8–72.1)	0.187
HOMA-IR^*∗*^	1.3 ± 0.8	1.3 ± 1.0	0.976
HOMA-*β*^*∗*^	3.9 ± 1.0	3.6 ± 0.9	0.179

^*∗*^Natural logarithm.

## Data Availability

The datasets generated and/or analyzed during the current study are available from the corresponding author on reasonable request.
